# Supplementary material for: Integrated analysis of genomic and transcriptomic data for the discovery of splice-associated variants in cancer
Source: Nat Commun. 2023 Mar 22;14:1589. doi: 10.1038/s41467-023-37266-6 (PMC10033906; doi:10.1038/s41467-023-37266-6)
Supplement: Supplementary file 1 — Supplementary Information [file 41467_2023_37266_MOESM1_ESM.pdf]

Supplemental Figures

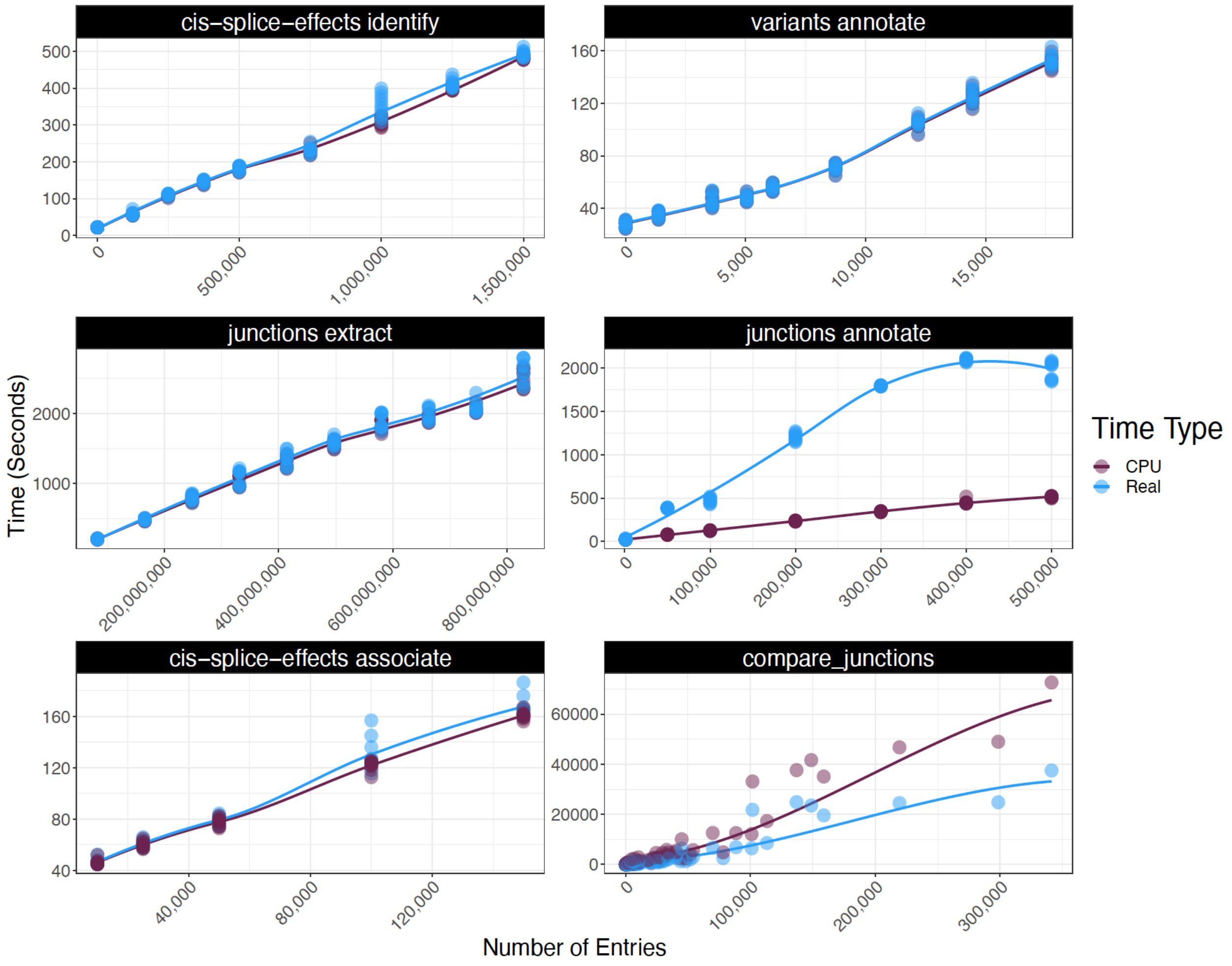

Supplementary Figure 1. Computational efficiency of Regtools

The total CPU time (System Time + User Time) and real-time are plotted against the number of entries processed for each available RegTools function using ten replicates. For the cis-splice-effects identify/cis-splice-effects associate/variants annotate workflows, the number of entries corresponds to the number of somatic variants. In contrast, the number of entries in the junctions extract/junctions annotate/compare_junctions workflows corresponds to the number of reads processed from a downsampled BAM file, the number of junctions processed, and the number of candidate variant junction pairings processed, respectively. For the statistical filtering performed by the supplementary compare_junctions pipeline, candidate variant junction pairings were compared across the number of samples in that cohort, with the largest being 1,022 samples that comprise our BRCA cohort. LOESS curves are fitted onto each plot. Source data are provided as a Source Data file.

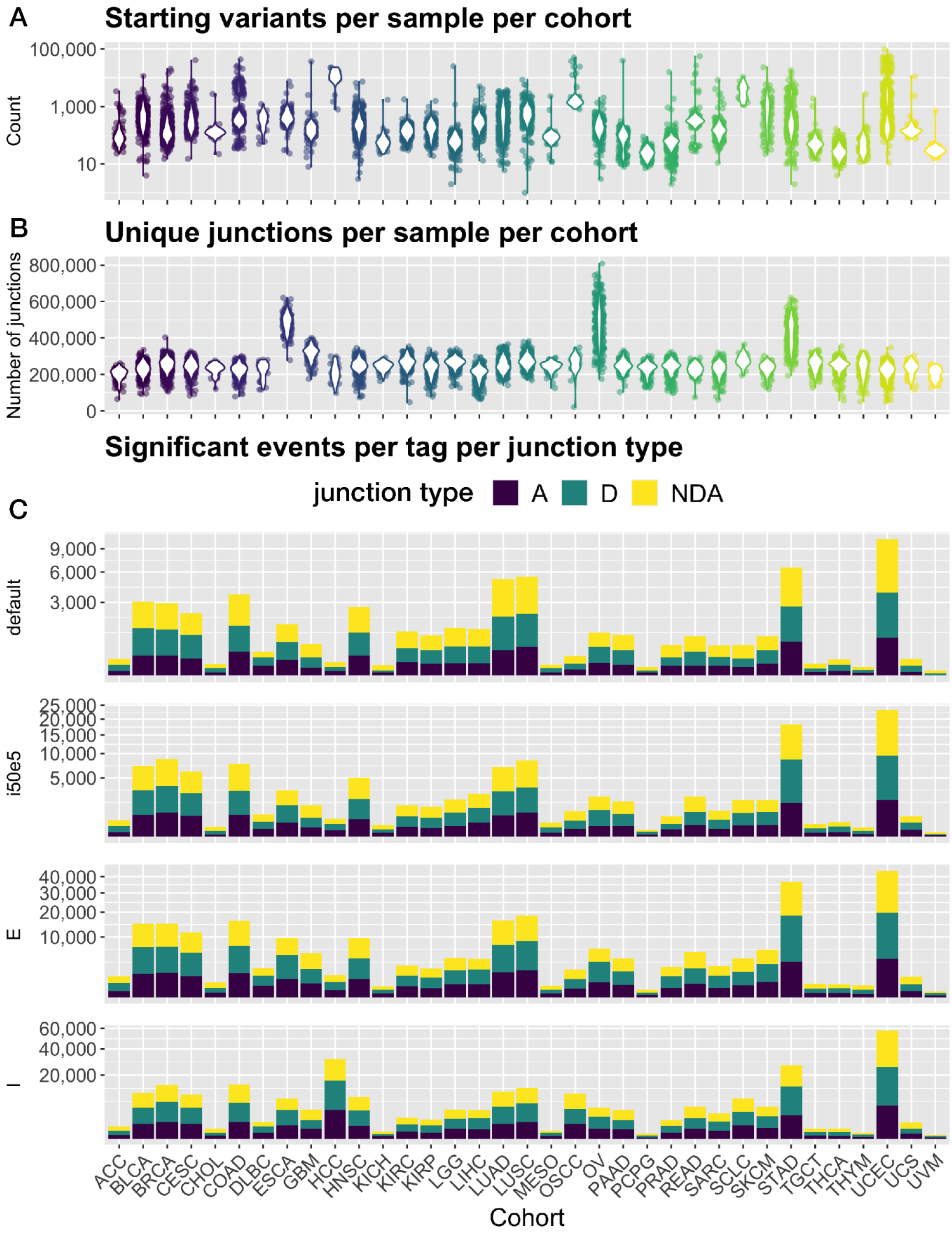
A
Starting variants per sample per cohort
Count
100,000
1,000
10
B
Unique junctions per sample per cohort
Number of junctions
800,000
600,000
400,000
200,000
0
Significant events per tag per junction type
junction type
A
D
NDA
C
default
9,000
6,000
3,000
i50e5
25,000
20,000
15,000
10,000
5,000
E
40,000
30,000
20,000
10,000
I
60,000
40,000
20,000
ACC BLCA BRCA CESC CHOL COAD DLBC ESCA GBM HCC HNSC KICH KIRC KIRP LGG LIHC LUAD LUSC MESO OSCC OV PAAD PCPG PRAD READ SARC SCLC SKCM STAD TGCT THCA THYM UCEC UCS UVM
Cohort

Supplementary Figure 2. Overview of input data considered and significant events identified by RegTools for each tumor type.

A) Summary of initial variants considered for analysis by RegTools per sample per tumor cohort. Each sample's variant count is plotted, and violin plots are overlaid for each cohort. B) Summary of unique exon-exon junction observations for each sample. Each sample's unique junction count is plotted, and violin plots are overlaid for each cohort. C) Summary of significant junction types for each cohort for each of the splice variant window parameters that were used in this analysis. Source data are provided as a Source Data file.

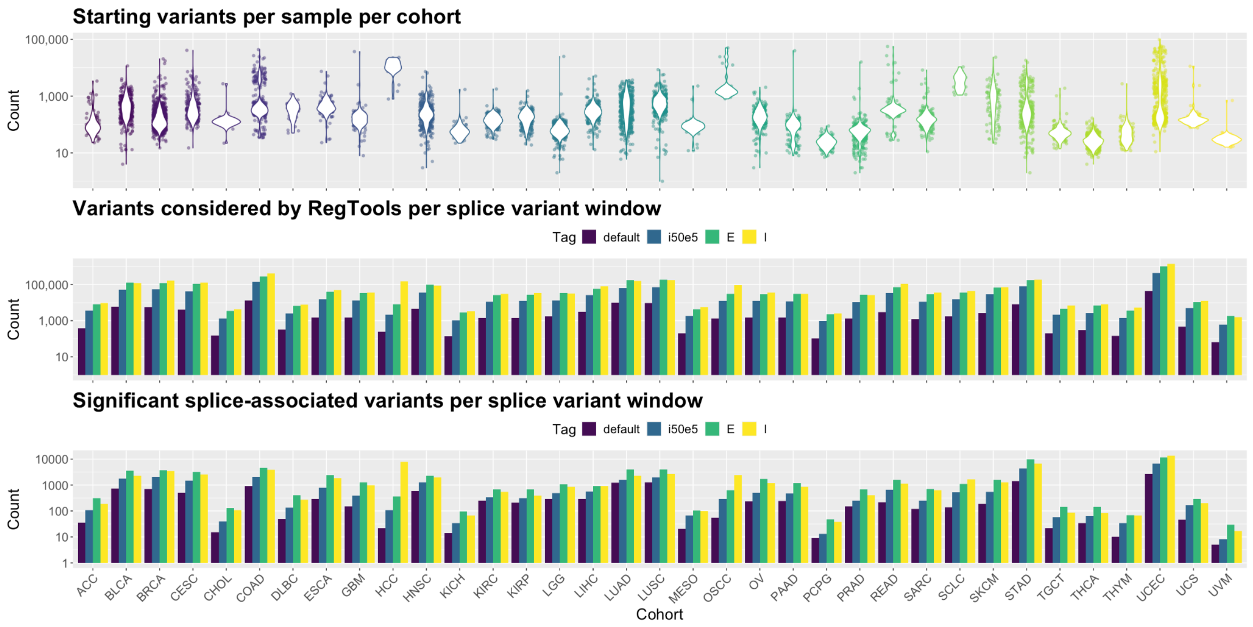

Supplementary Figure 3. Summary of variants analyzed by RegTools in each tumor cohort

Summary of the starting number of high-quality variants per sample, the number of initial variants considered for analysis by RegTools for each variant window used per tumor cohort, and the number of significant variants for each variant window used per tumor cohort. Source data are provided as a Source Data file.

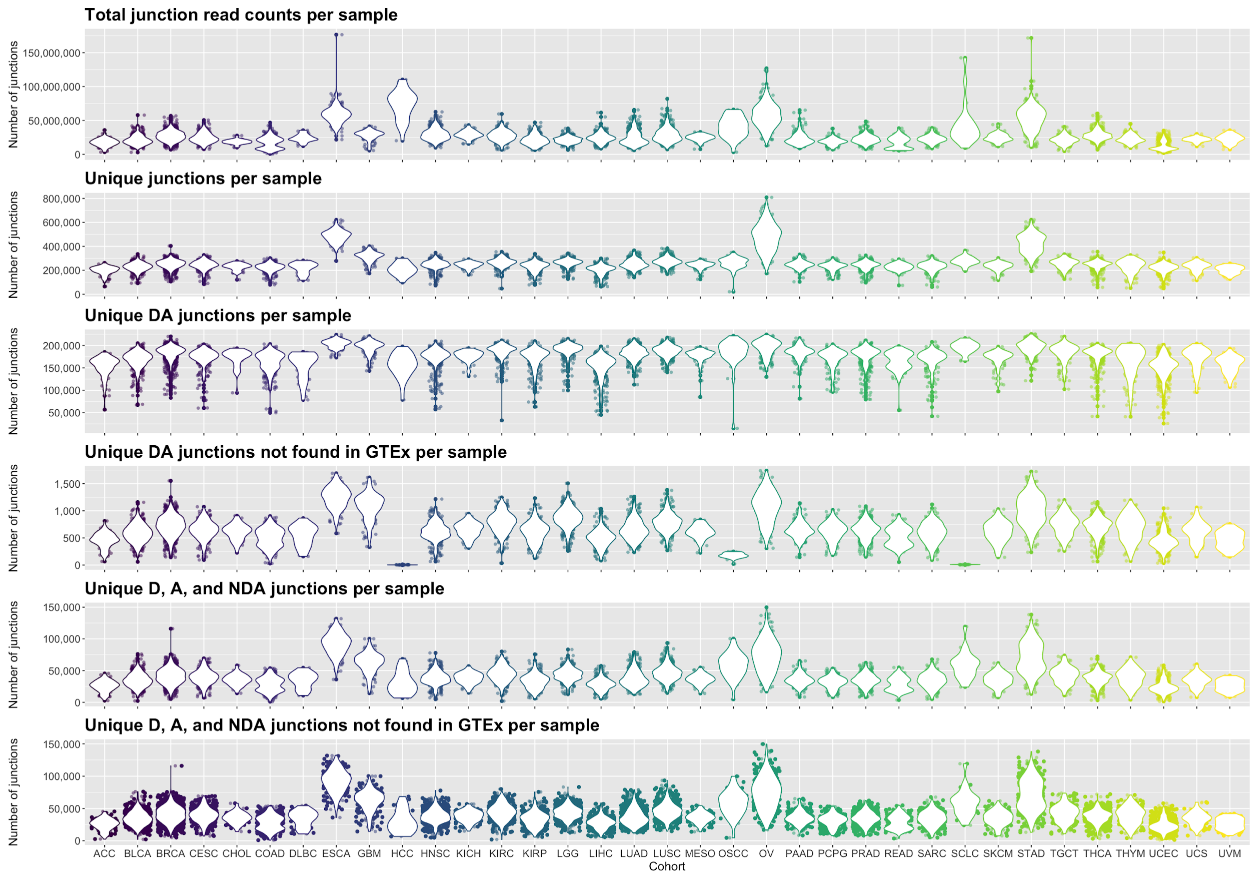

Supplementary Figure 4. Diversity of alternative splicing patterns across cancer types.

Summary of the total junction read counts, unique junctions (all types), unique known (DA) junctions, unique known (DA) junctions not found in GTEx, unique D, A, NDA junctions, and unique D, A, NDA junctions not found in GTEx per sample per cohort. These results were obtained using the all exonic (-E) and all intronic (-I) splice variant window parameters. Source data are provided as a Source Data file.

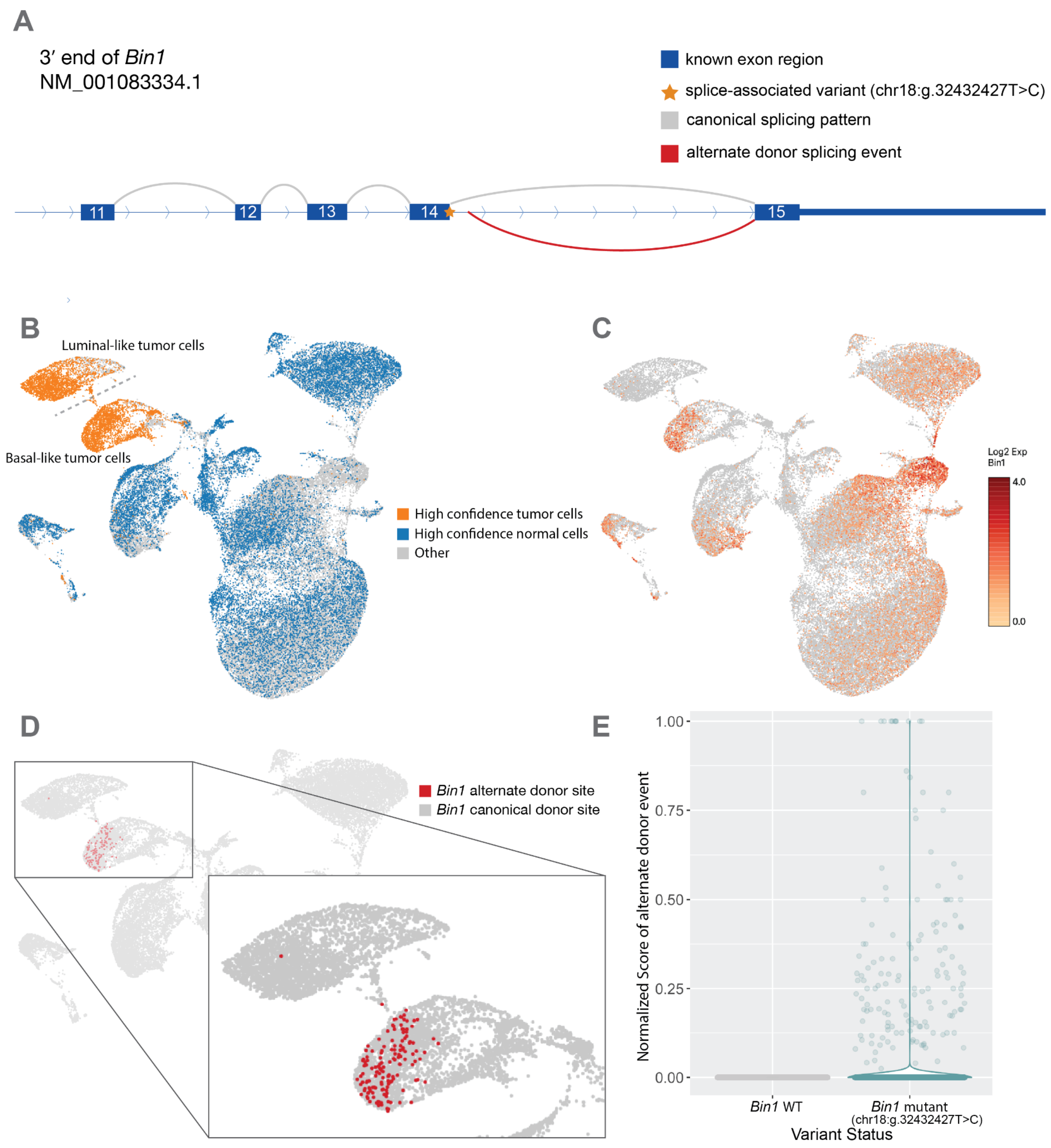

Supplementary Figure 5: Intronic SNV in *Bin1* associated with alternate donor usage by scRNA-seq analysis.

A) Schematic of a single nucleotide variant (mm10, chr18:g.32432427T>C, c.1516+2 position of intron 14 of transcript NM_001083334.1) within an intron of *Bin1*. This variant is significantly associated with an alternate donor event causing the formation of a novel junction. This result was found using the default splice variant window parameter (i2e3). **B)** UMAP projection of

single cells from MCB6C organoid-derived tumors with high confidence tumor cells (orange) and high confidence normal cells (blue) highlighted. **C)** UMAP projection of single cells from MCB6C organoid-derived tumors overlaid with Log2 expression of Bin1. **D)** Zoomed view of cells containing the Bin1 alternate donor event. **E)** Violin plot comparing the normalized junction score of the novel alternate donor event in cells with and without the variant. Source data are provided as a Source Data file.

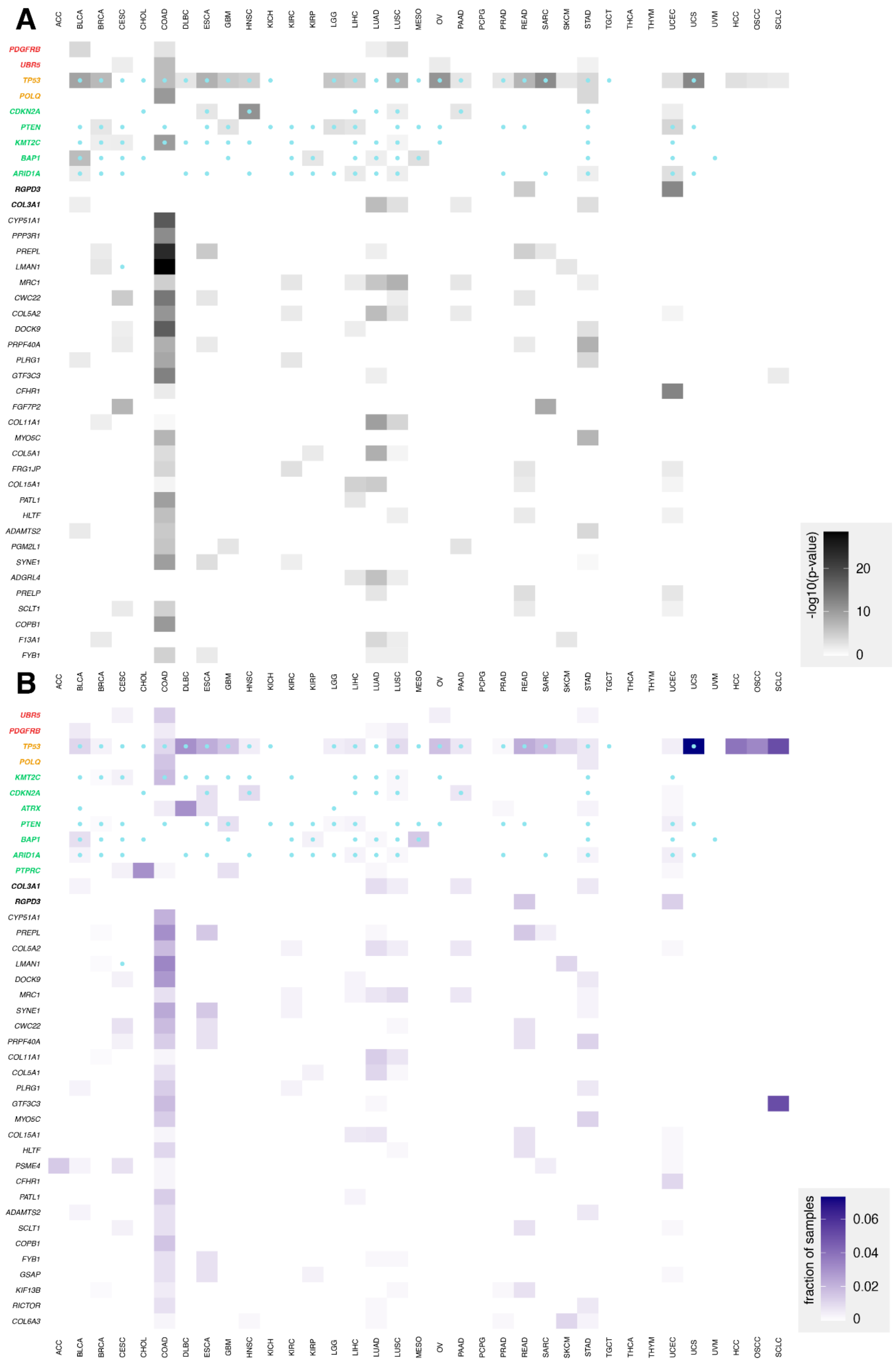
A
B
ACC BLCA BRCA CESC CHOL COAD DLBC ESCA GBM HNSC KICH KIRC KIRP LGG LIHC LUAD LUSC MESO OV PAAD PCPG PRAD READ SARC SKCM STAD TGCT THCA THYM UCEC UCS UVM HCC OSCC SCLC
PDGFRB UBR5 TP53 POLQ CDKN2A PTEN KMT2C BAP1 ARID1A RGPD3 COL3A1 CYP51A1 PPP3R1 PREPL LMAN1 MRC1 CWC22 COL5A2 DOCK9 PRPF40A PLRG1 GTF3C3 CFHR1 FGF7P2 COL11A1 MYO5C COL5A1 FRG1JP COL15A1 PATL1 HLTF ADAMTS2 PGM2L1 SYNE1 ADGRL4 PRELP SCLT1 COPB1 F13A1 FYB1
-log10(p-value)
0
10
20
UBR5 PDGFRB TP53 POLQ KMT2C CDKN2A ATRX PTEN BAP1 ARID1A PTPRC COL3A1 RGPD3 CYP51A1 PREPL COL5A2 LMAN1 DOCK9 MRC1 SYNE1 CWC22 PRPF40A COL11A1 COL5A1 PLRG1 GTF3C3 MYO5C COL15A1 HLTF PSME4 CFHR1 PATL1 ADAMTS2 SCLT1 COPB1 FYB1 GSAP KIF13B RICTOR COL6A3
fraction of samples
0
0.02
0.04
0.06

Supplementary Figure 6. Pan-cancer analysis of cohorts from TCGA and MGI reveals genes recurrently disrupted by variants that promote splicing of particular canonical junctions

Results of analysis for recurrently disrupted genes in each TCGA and MGI cohort. **A)** Rows correspond to the 40 most frequently recurring genes, as ranked by binomial p-value (See Methods, *Identification of genes with recurrent splice-associated variants*). Genes are clustered by whether they were annotated by the CGC as an oncogene (red), an oncogene and tumor suppressor gene (yellow), a tumor suppressor gene (green), or another type of cancer-relevant gene (black, bold). Shading corresponds to −log10(p-value) and columns represent cancer types. Blue marks within cells indicate that the gene was annotated by CHASMplus as a driver within a given TCGA cohort. **B)** Rows correspond to the 40 most frequently recurring genes, as ranked by the fraction of samples. Shading corresponds to the fraction of samples and columns represent cancer types. Blue dots within cells indicate that the gene was annotated by CHASMplus as a driver within a given TCGA cohort. These results were obtained using the default splice variant window parameter (i2e3). Source data are provided as a Source Data file.

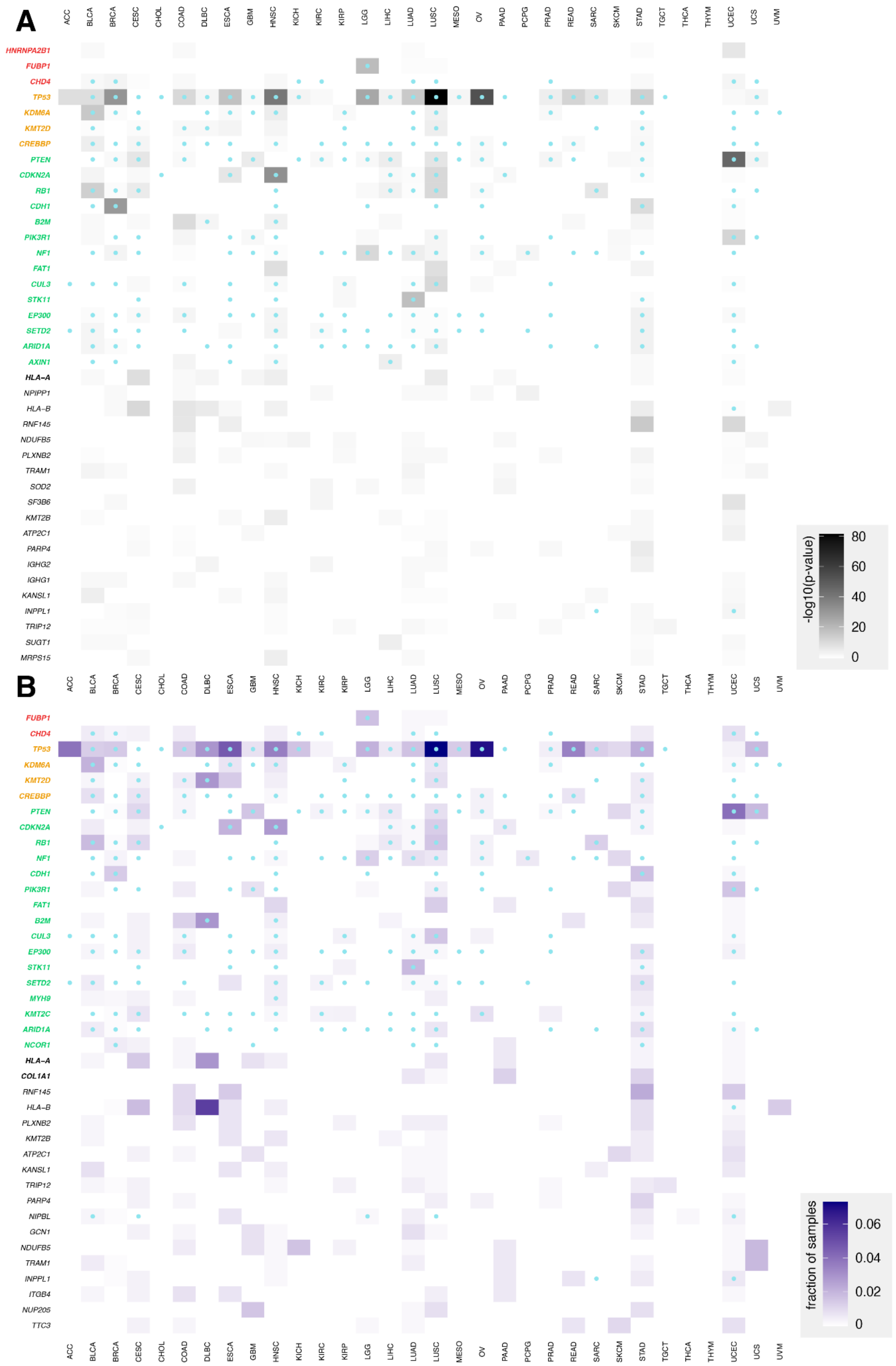
A
B
HNRNPA2B1
FUBP1
CHD4
TP53
KDM6A
KMT2D
CREBBP
PTEN
CDKN2A
RB1
CDH1
B2M
PIK3R1
NF1
FAT1
CUL3
STK11
EP300
SETD2
ARID1A
AXIN1
HLA-A
NPIPP1
HLA-B
RNF145
NDUFB5
PLXNB2
TRAM1
SOD2
SF3B6
KMT2B
ATP2C1
PARP4
IGHG2
IGHG1
KANSL1
INPPL1
TRIP12
SUGT1
MRPS15
MYH9
KMT2C
NCOR1
COL1A1
NIPBL
GCN1
ITGB4
NUP205
TTC3
ACC
BLCA
BRCA
CESC
CHOL
COAD
DLBC
ESCA
GBM
HNSC
KICH
KIRC
KIRP
LGG
LIHC
LUAD
LUSC
MESO
OV
PAAD
PCPG
PRAD
READ
SARC
SKCM
STAD
TGCT
THCA
THYM
UCEC
UCS
UVM
-log10(p-value)
0
20
40
60
80
fraction of samples
0
0.02
0.04
0.06

Supplementary Figure 7. TCGA pan-cancer analysis reveals genes recurrently disrupted by variants that cause non-canonical splicing patterns

Results of analysis for recurrently disrupted genes in each TCGA cohort. **A)** Rows correspond to the 40 most frequently recurring genes, as ranked by binomial p-value (See Methods, *Identification of genes with recurrent splice-associated variants*). Genes are clustered by whether they were annotated by the CGC as an oncogene (red), an oncogene and tumor suppressor gene (yellow), a tumor suppressor gene (green), or another type of cancer-relevant gene (black, bold). Shading corresponds to −log10(p-value) and columns represent cancer types. Blue marks within cells indicate that the gene was annotated by CHASMplus as a driver within a given TCGA cohort. **B)** Rows correspond to the 40 most frequently recurring genes, as ranked by the fraction of samples. Shading corresponds to the fraction of samples and columns represent cancer types. Blue dots within cells indicate that the gene was annotated by CHASMplus as a driver within a given TCGA cohort. These results were obtained using the default splice variant window parameter (i2e3). Source data are provided as a Source Data file.

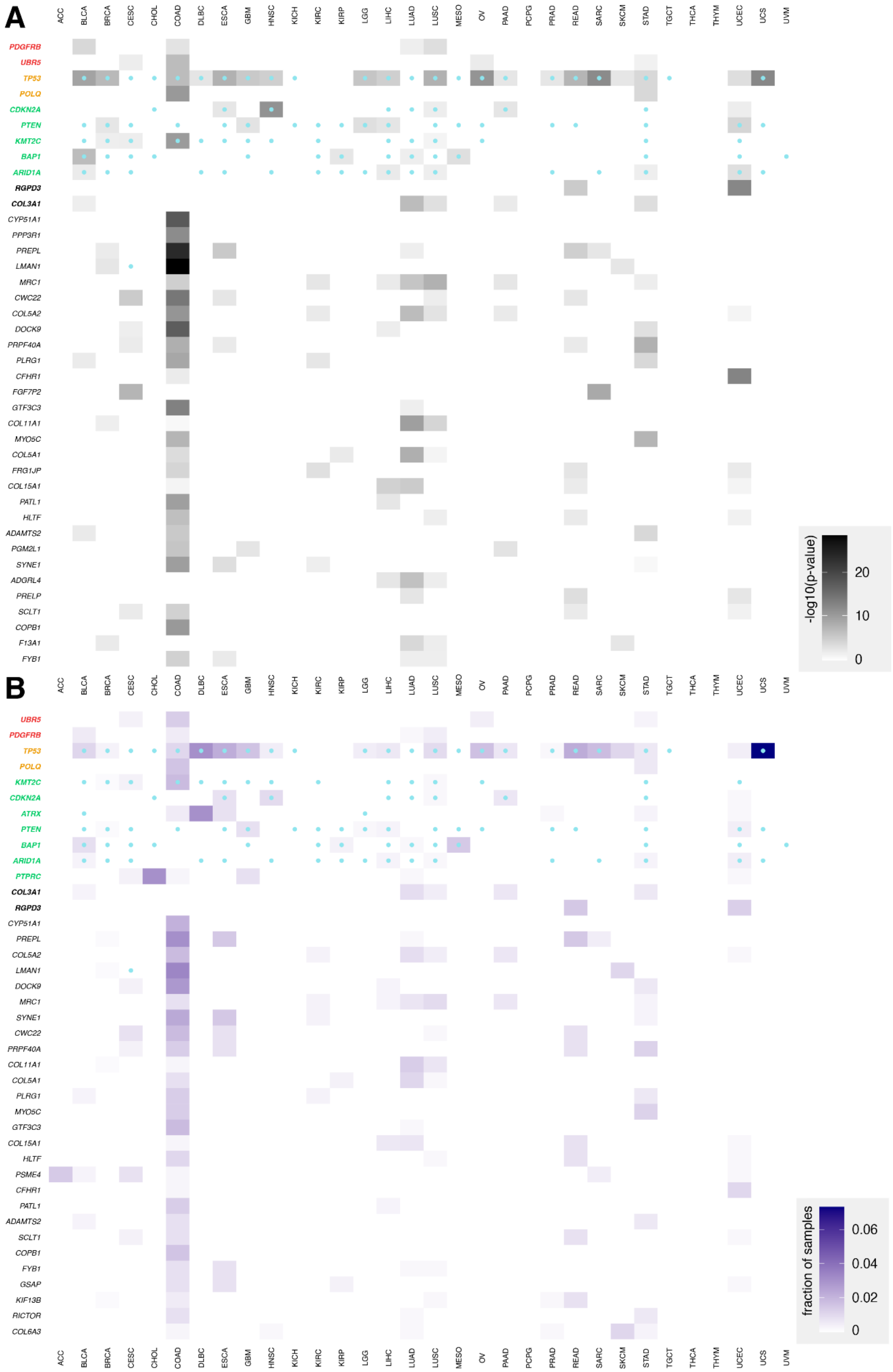
A
ACC BLCA BRCA CESC CHOL COAD DLBC ESCA GBM HNSC KICH KIRC KIRP LGG LIHC LUAD LUSC MESO OV PAAD PCPG PRAD READ SARC SKCM STAD TGCT THCA THYM UCEC UCS UVM
PDGFRB
UBR5
TP53
POLQ
CDKN2A
PTEN
KMT2C
BAP1
ARID1A
RGPD3
COL3A1
CYP51A1
PPP3R1
PREPL
LMAN1
MRC1
CWC22
COL5A2
DOCK9
PRPF40A
PLRG1
CFHR1
FGF7P2
GTF3C3
COL11A1
MYO5C
COL5A1
FRG1JP
COL15A1
PATL1
HLTF
ADAMTS2
PGM2L1
SYNE1
ADGRL4
PRELP
SCLT1
COPB1
F13A1
FYB1
-log10(p-value)
20
10
0
B
UBR5
PDGFRB
TP53
POLQ
KMT2C
CDKN2A
ATRX
PTEN
BAP1
ARID1A
PTPRC
COL3A1
RGPD3
CYP51A1
PREPL
COL5A2
LMAN1
DOCK9
MRC1
SYNE1
CWC22
PRPF40A
COL11A1
COL5A1
PLRG1
MYO5C
GTF3C3
COL15A1
HLTF
PSME4
CFHR1
PATL1
ADAMTS2
SCLT1
COPB1
FYB1
GSAP
KIF13B
RICTOR
COL6A3
fraction of samples
0.06
0.04
0.02
0

Supplementary Figure 8. TCGA pan-cancer analysis reveals genes recurrently disrupted by variants that promote splicing of particular canonical junctions

Results of analysis for recurrently disrupted genes in each TCGA cohort. **A)** Rows correspond to the 40 most frequently recurring genes, as ranked by binomial p-value (See Methods, *Identification of genes with recurrent splice-associated variants*). Genes are clustered by whether they were annotated by the CGC as an oncogene (red), an oncogene and tumor suppressor gene (yellow), a tumor suppressor gene (green), or another type of cancer-relevant gene (black, bold). Shading corresponds to −log10(p-value) and columns represent cancer types. Blue marks within cells indicate that the gene was annotated by CHASMplus as a driver within a given TCGA cohort. **B)** Rows correspond to the 40 most frequently recurring genes, as ranked by the fraction of samples. Shading corresponds to the fraction of samples and columns represent cancer types. Blue dots within cells indicate that the gene was annotated by CHASMplus as a driver within a given TCGA cohort. These results were obtained using the default splice variant window parameter (i2e3). Source data are provided as a Source Data file.

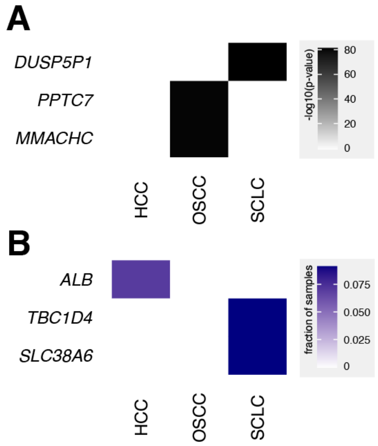

Supplementary Figure 9. Analysis of MGI cohorts reveals genes recurrently disrupted by variants that cause non-canonical splicing patterns

Results of analysis for recurrently disrupted genes in each MGI cohort. **A)** Rows correspond to the 40 most frequently recurring genes, as ranked by binomial p-value (See Methods, *Identification of genes with recurrent splice-associated variants*). Genes are clustered by whether they were annotated by the CGC as an oncogene (red), an oncogene and tumor suppressor gene (yellow), a tumor suppressor gene (green), or another type of cancer-relevant gene (black, bold). Shading corresponds to −log10(p-value) and columns represent cancer types. **B)** Rows correspond to the 40 most frequently recurring genes, as ranked by the fraction of samples. Shading corresponds to the fraction of samples and columns represent cancer types. These results were obtained using the default splice variant window parameter (i2e3). Source data are provided as a Source Data file.

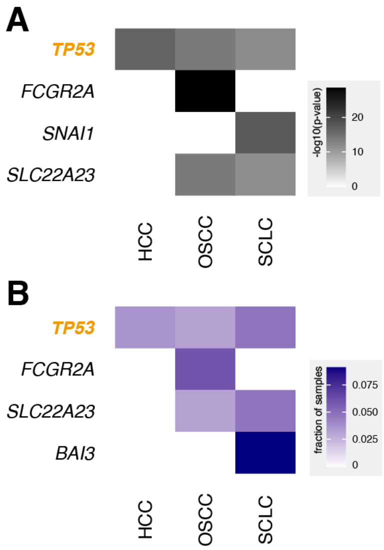

Supplementary Figure 10. Analysis of MGI cohorts reveals genes recurrently disrupted by variants that promote splicing of particular canonical junctions

Results of analysis for recurrently disrupted genes in each MGI cohort. **A)** Rows correspond to the 4 most frequently recurring genes, as ranked by binomial p-value (See Methods, *Identification of genes with recurrent splice-associated variants*). Genes are clustered by whether they were annotated by the CGC as an oncogene (red), an oncogene and tumor suppressor gene (yellow), a tumor suppressor gene (green), or another type of cancer-relevant gene (black, bold). Shading corresponds to −log10(p-value) and columns represent cancer types. **B)** Rows correspond to the 4 most frequently recurring genes, as ranked by the fraction of samples. Shading corresponds to the fraction of samples and columns represent cancer types. These results were obtained using the default splice variant window parameter (i2e3). Source data are provided as a Source Data file.

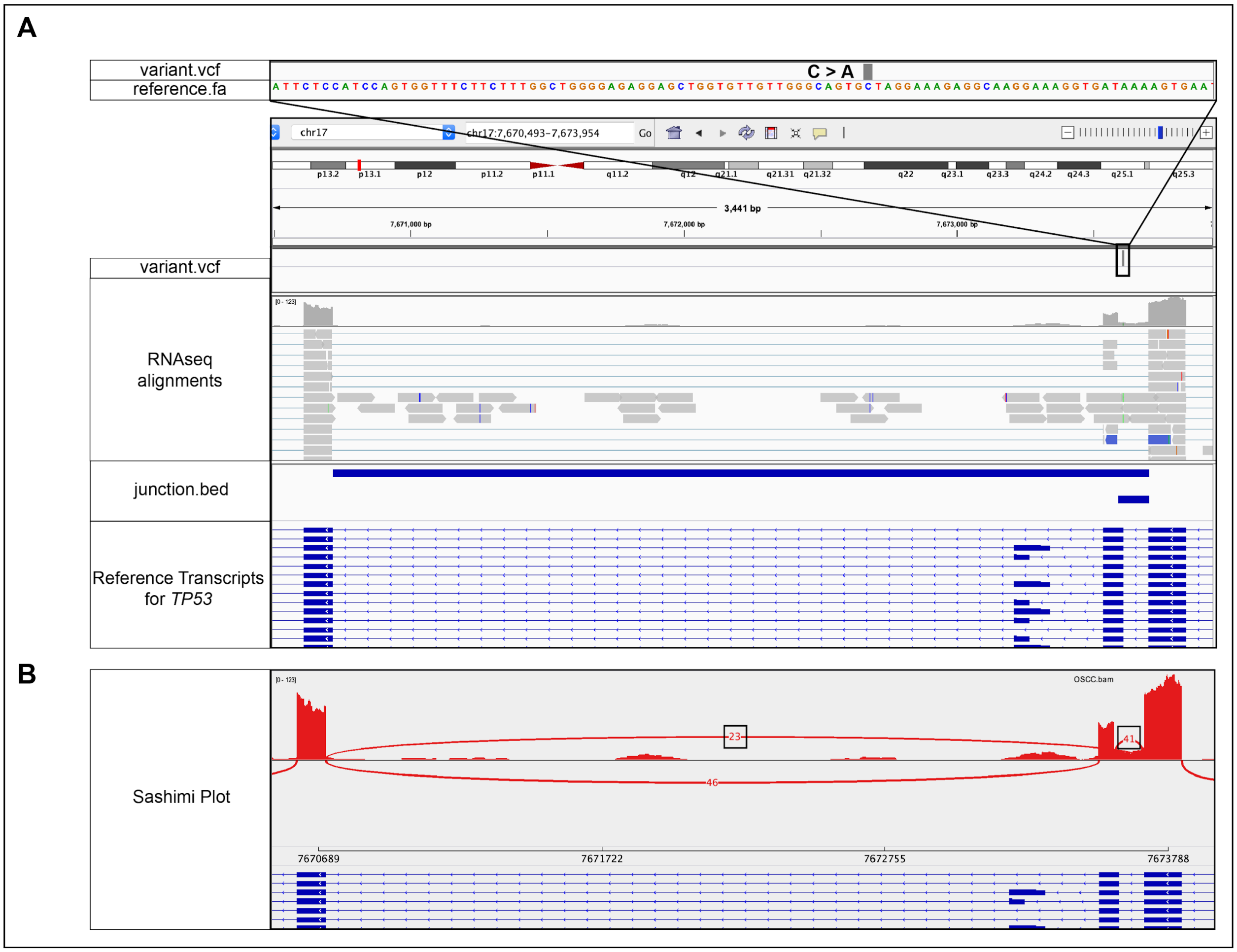

Supplementary Figure 11: Intronic SNV in *TP53* associated with exon skipping and alternative donor usage.

A) IGV snapshot of a single nucleotide variant (GRCh38, chr17:g.7673609C>A, NM_000546.5) within an intron of *TP53* in an OSCC sample. This variant is associated with skipping of exon 9 (23 reads of support) and use of an alternate acceptor site (41 reads of support). This result was found using the default splice variant window parameter (i2e3). **B)** Sashimi plot visualization of the novel junction.

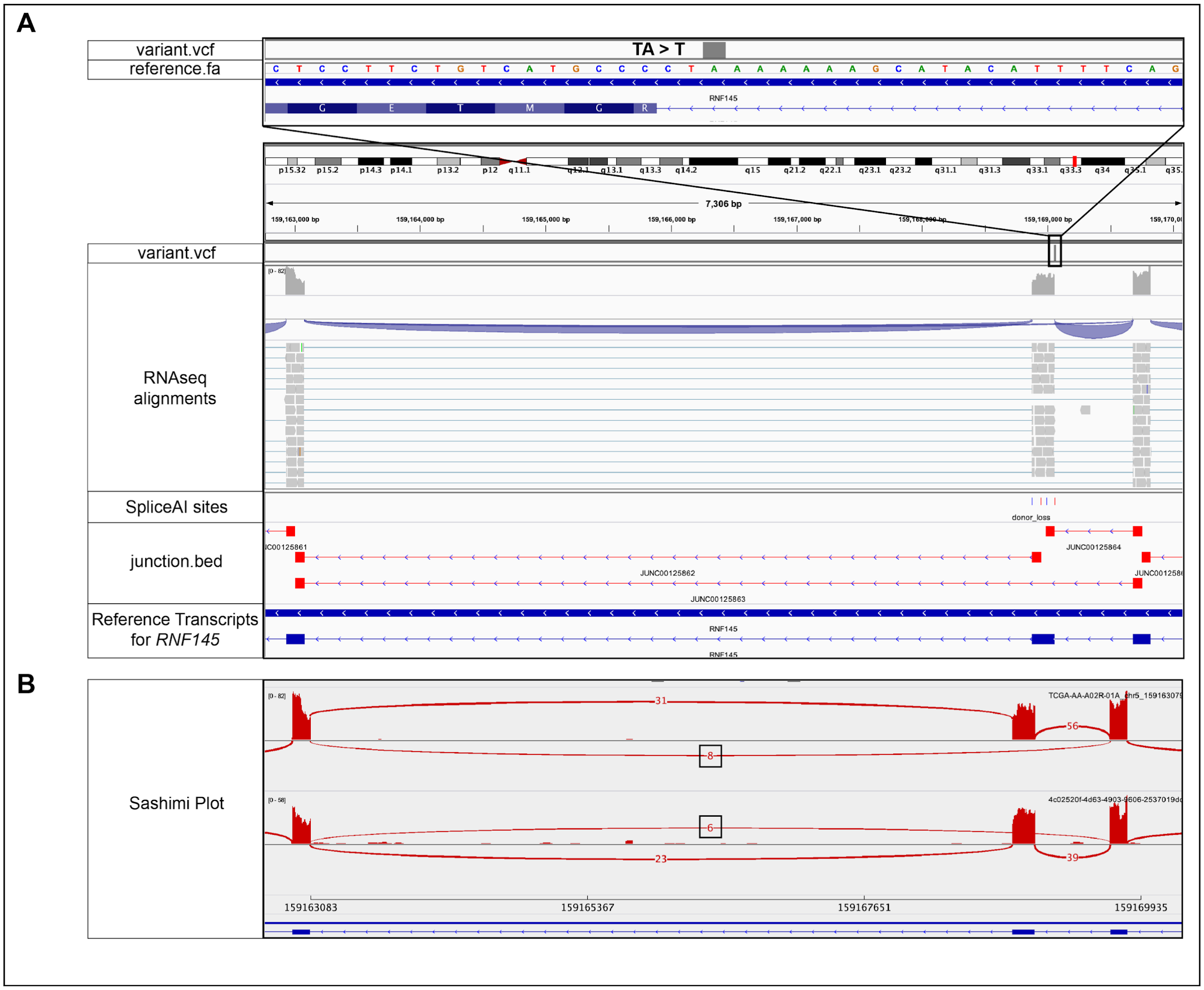

Supplementary Figure 12: Intronic deletion in *RNF145* associated with skipping of exon 8.

A) IGV snapshot of a single nucleotide variant (GRCh38, chr5:g.159169058delA, NM_001199383.2) within an intron of *RNF145* in COAD samples. This variant is associated with an exon skipping event with 8 and 6 reads of support for the samples shown. This result was found using the default splice variant window parameter (i2e3). **B)** Sashimi plot visualization of the novel junction.

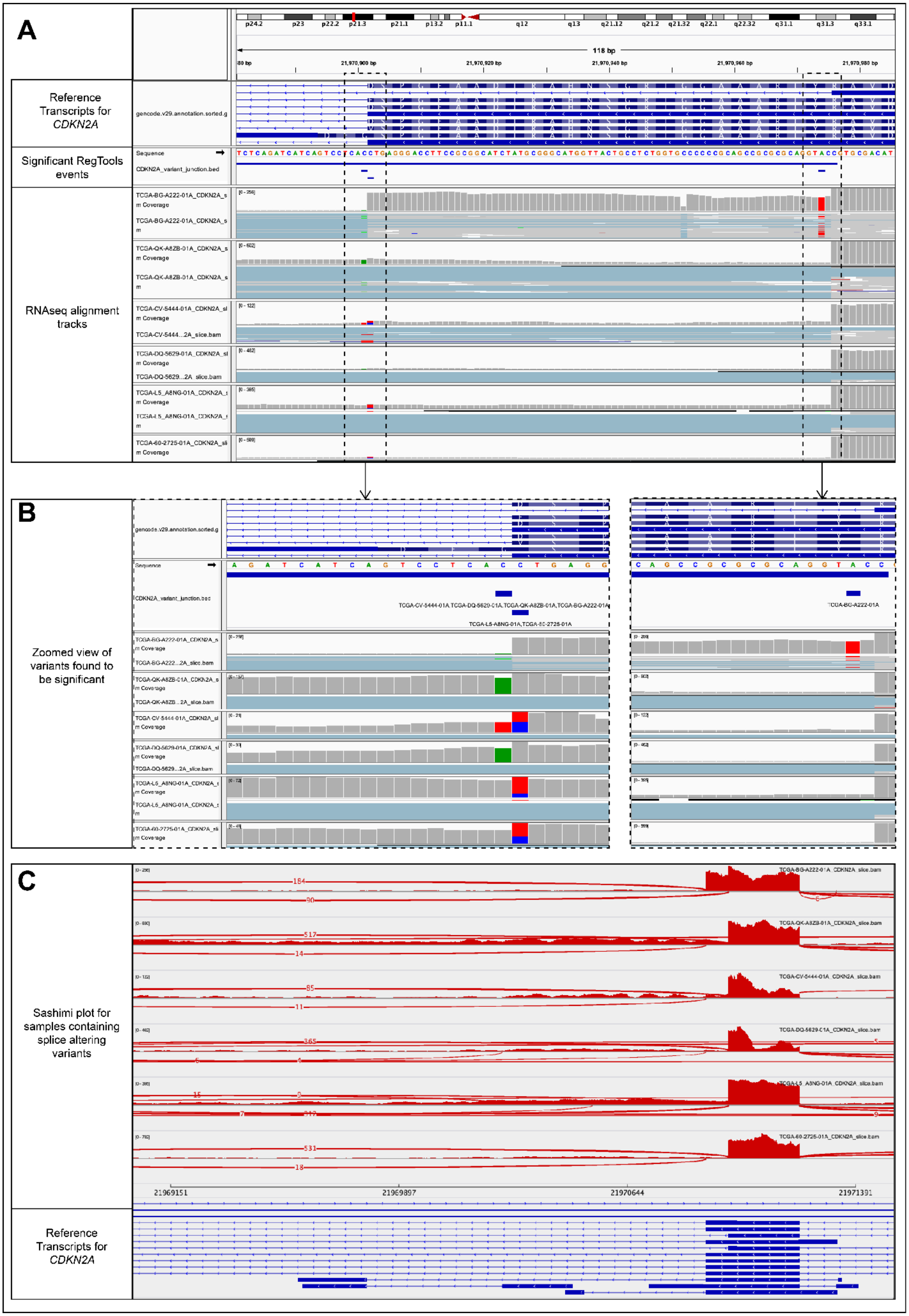
A
Reference Transcripts for *CDKN2A*
Significant RegTools events
RNAseq alignment tracks
118 bp
gencode.v29.annotation.sorted.g
Sequence
CDKN2A_variant_junction.bed
B
Zoomed view of variants found to be significant
TCGA-CV-5444-01A,TCGA-DQ-5629-01A,TCGA-QK-A8ZB-01A,TCGA-BG-A222-01A
TCGA-L5-A8NG-01A,TCGA-60-2725-01A
TCGA-BG-A222-01A
C
Sashimi plot for samples containing splice altering variants
21969151
21969897
21970644
21971391
Reference Transcripts for *CDKN2A*

Supplementary Figure 13: Several SNVs in *CDKN2A* associated with alternate donor usage leading to an alternate known junction that may cause C-terminus modification.

A) IGV snapshot of three variant positions in *CDKN2A* found to be associated with usage of an alternate donor site within exon 2 that leads to the formation of an alternate known junction included in an alternate transcript, ENST00000579122.1. This result was found using the default splice variant window parameter (i2e3) for known (DA) junctions. **B)** Zoomed in view of the variants identified by RegTools that are associated with alternate donor usage. Two of these variant positions flank the donor site that is no longer being used. **C)** Sashimi plot visualizations for samples containing the identified variants that show alternate donor usage.

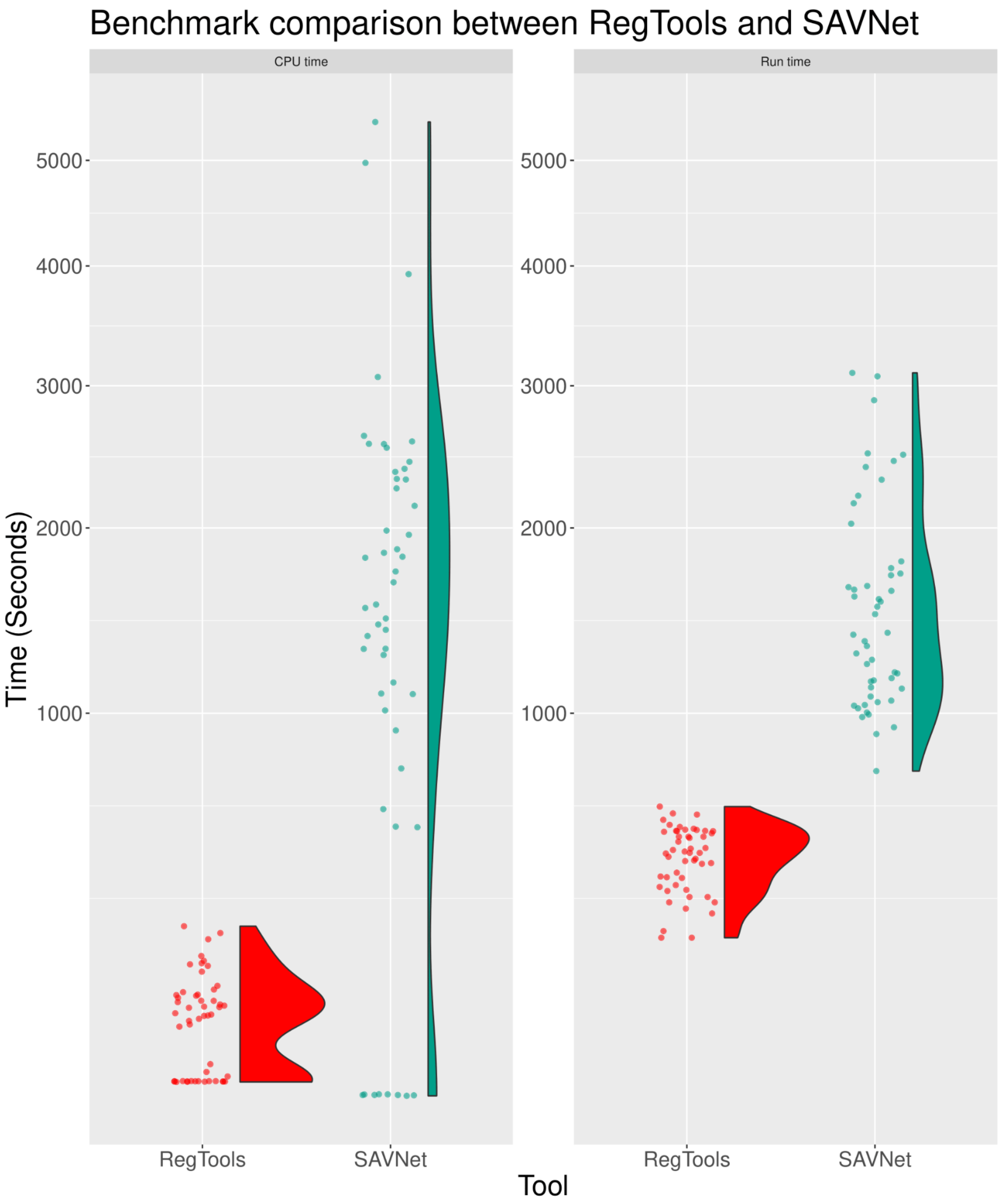

Supplementary Figure 14. Benchmarking of RegTools and SAVNet

The total CPU time (System Time + User Time) and real-time are plotted against both RegTools and SAVNet for fifty LUAD samples from TCGA. Each sample is represented as a point and a half violin plot is plotted alongside the dot plot to show the distribution of runtimes. These results were obtained using the default splice variant window parameter (i2e3). Source data are provided as a Source Data file.
