# Supplementary material for: Integrated analysis of genomic and transcriptomic data for the discovery of splice-associated variants in cancer
Source: Nat Commun. 2023 Mar 22;14:1589. doi: 10.1038/s41467-023-37266-6 (PMC10033906; doi:10.1038/s41467-023-37266-6)
Supplement: Supplementary file 2 — Description of Additional Supplementary Files [file 41467_2023_37266_MOESM2_ESM.docx]

# Supplementary Data Files:

**Supplementary Data File 1**: Candidate variant junction pairings for D, A, NDA junctions (GRCh38 samples)

**Supplementary Data File 2**: Candidate variant junction pairings for D, A, NDA junctions (GRCh37 samples)

**Supplementary Data File 3**: Candidate variant junction pairings for DA junctions (GRCh38 samples)

**Supplementary Data File 4**: Candidate variant junction pairings for DA junctions (GRCh37 samples)

**Supplementary Data File 5**: Validation of MiSplice mini-gene assay findings using RegTools

**Supplementary Data File 6**: MutSpliceDB splice-associated variants validated using RegTools

**Supplementary Data File 7**: RegTools analysis for GBM/Brain metastases samples with multi-sector sequencing

**Supplementary Data File 8**: RegTools analysis for SCLC samples with treatment-naive and recurrence timepoints

**Supplementary Data File 9**: RegTools analysis of HCC1395 cell line with correlated Oxford Nanopore sequencing

**Supplementary Data File 10**: Recurrence analysis for D, A, NDA junctions in the default splice variant window

**Supplementary Data File 11**: Recurrence analysis for D, A, NDA junctions in the i50e5 splice variant window

**Supplementary Data File 12**: Recurrence analysis for D, A, NDA junctions in the E (all exonic) splice variant window

**Supplementary Data File 13**: Recurrence analysis for D, A, NDA junctions in the I (all intronic) splice variant window

**Supplementary Data File 14**: Recurrence analysis for DA junctions in the default splice variant window

**Supplementary Data File 15**: GTEx tissue type mapping to TCGA cohorts

**Supplementary Figures File**: Supplementary figures 1-14
