# Supplementary material for: Integrated analysis of genomic and transcriptomic data for the discovery of splice-associated variants in cancer
Source: Nat Commun. 2023 Mar 22;14:1589. doi: 10.1038/s41467-023-37266-6 (PMC10033906; doi:10.1038/s41467-023-37266-6)
Supplement: Supplementary file 18 — Reporting Summary [file 41467_2023_37266_MOESM18_ESM.pdf]

nature portfolio

Corresponding author(s): Malachi Griffith
Last updated by author(s): Jun 27, 2022

Reporting Summary

Nature Portfolio wishes to improve the reproducibility of the work that we publish. This form provides structure for consistency and transparency in reporting. For further information on Nature Portfolio policies, see our Editorial Policies and the Editorial Policy Checklist.

Statistics

For all statistical analyses, confirm that the following items are present in the figure legend, table legend, main text, or Methods section.

n/a	Confirmed	
☐	☒	The exact sample size (*n*) for each experimental group/condition, given as a discrete number and unit of measurement
☐	☒	A statement on whether measurements were taken from distinct samples or whether the same sample was measured repeatedly
☐	☒	The statistical test(s) used AND whether they are one- or two-sided<br>*Only common tests should be described solely by name; describe more complex techniques in the Methods section.*
☐	☒	A description of all covariates tested
☐	☒	A description of any assumptions or corrections, such as tests of normality and adjustment for multiple comparisons
☐	☒	A full description of the statistical parameters including central tendency (e.g. means) or other basic estimates (e.g. regression coefficient) AND variation (e.g. standard deviation) or associated estimates of uncertainty (e.g. confidence intervals)
☐	☒	For null hypothesis testing, the test statistic (e.g. *F*, *t*, *r*) with confidence intervals, effect sizes, degrees of freedom and *P* value noted<br>*Give P values as exact values whenever suitable.*
☒	☐	For Bayesian analysis, information on the choice of priors and Markov chain Monte Carlo settings
☒	☐	For hierarchical and complex designs, identification of the appropriate level for tests and full reporting of outcomes
☒	☐	Estimates of effect sizes (e.g. Cohen's *d*, Pearson's *r*), indicating how they were calculated

Our web collection on statistics for biologists contains articles on many of the points above.

Software and code

Policy information about availability of computer code

Data collection	For TCGA data, we downloaded variant calls and RNA-sequencing data from the Genomic Data Commons (GDC; https://portal.gdc.cancer.gov/) as vcf and BAM files. All other data was sequenced and processed at Washington University in St. Louis.
Data analysis	RegTools is open source (MIT license) and available at https://github.com/griffithlab/regtools/. All scripts used in the analyses presented here are also provided. For ease of use, a Docker container has been created with RegTools, SpliceAI, R, and Python 3 installed (https://hub.docker.com/r/griffithlab/regtools/). This Docker container allows a user to run the workflow we outline at https://regtools.readthedocs.io/en/latest/workflow/. Docker is an open-source software platform that enables applications to be readily installed and run on any system. The availability of RegTools with all its dependencies as a Docker container also facilitates the integration of the RegTools software into workflow pipelines that support Docker images.

For manuscripts utilizing custom algorithms or software that are central to the research but not yet described in published literature, software must be made available to editors and reviewers. We strongly encourage code deposition in a community repository (e.g. GitHub). See the Nature Portfolio guidelines for submitting code & software for further information.

Data

Policy information about availability of data

All manuscripts must include a data availability statement. This statement should provide the following information, where applicable:

- Accession codes, unique identifiers, or web links for publicly available datasets
- A description of any restrictions on data availability
- For clinical datasets or third party data, please ensure that the statement adheres to our policy

Sequence data for each cohort analyzed in this study are available through dbGaP at the following accession IDs: phs000178 [https://www.ncbi.nlm.nih.gov/projects/gap/cgi-bin/study.cgi?study_id=phs000178] for TCGA cohorts, phs001106 [https://www.ncbi.nlm.nih.gov/projects/gap/cgi-bin/study.cgi?study_id=phs001106] for HCC, phs001049 [https://www.ncbi.nlm.nih.gov/projects/gap/cgi-bin/study.cgi?study_id=phs001049] for SCLC, phs001623 [https://www.ncbi.nlm.nih.gov/projects/gap/cgi-bin/study.cgi?study_id=phs001623] for OSCC, and phs002612 [https://www.ncbi.nlm.nih.gov/projects/gap/cgi-bin/study.cgi?study_id=phs002612] for GBM/Brain metastases. Both short read and long read RNA sequencing data for the HCC1395 cell line are available in the Sequence Read Archive (SRA) under the following accession IDs: SRX278519 [https://www.ncbi.nlm.nih.gov/sra/SRX278519[accn]] and PRJNA934933 [https://www.ncbi.nlm.nih.gov/bioproject/PRJNA934933]. Single cell RNA expression data is available in the SRA at the accession: PRJNA934380 https://www.ncbi.nlm.nih.gov/bioproject/PRJNA934380. Statistically significant events for D, A, and NDA junctions across the four variant splicing windows used are available via Supplementary Files 1 and 2. Statistically significant events for DA junctions are available as Supplementary Files 3 and 4. Complete results of gene recurrence analysis are available as Supplementary Files 10-14. Source data are provided with this paper.

Human research participants

Policy information about studies involving human research participants and Sex and Gender in Research.

Reporting on sex and gender	N/A
Population characteristics	N/A
Recruitment	N/A
Ethics oversight	N/A

Note that full information on the approval of the study protocol must also be provided in the manuscript.

Field-specific reporting

Please select the one below that is the best fit for your research. If you are not sure, read the appropriate sections before making your selection.

☒ Life sciences ☐ Behavioural & social sciences ☐ Ecological, evolutionary & environmental sciences

For a reference copy of the document with all sections, see nature.com/documents/nr-reporting-summary-flat.pdf

Life sciences study design

All studies must disclose on these points even when the disclosure is negative.

Sample size	All samples from each study described were used.
Data exclusions	No data was excluded.
Replication	Not applicable because the findings are reported through existing data that is available upon request. Therefore, the results can be repeated and fully replicated using code provided.
Randomization	All data was included and there was no need for randomization of data as the presence of variants determined whether a sample was a case or a control.
Blinding	Not relevant to this study.

Reporting for specific materials, systems and methods

We require information from authors about some types of materials, experimental systems and methods used in many studies. Here, indicate whether each material, system or method listed is relevant to your study. If you are not sure if a list item applies to your research, read the appropriate section before selecting a response.

Materials & experimental systems

n/a	Involved in the study
☐	☒ Antibodies
☐	☒ Eukaryotic cell lines
☒	☐ Palaeontology and archaeology
☐	☒ Animals and other organisms
☒	☐ Clinical data
☒	☐ Dual use research of concern

Methods

n/a	Involved in the study
☒	☐ ChIP-seq
☒	☐ Flow cytometry
☒	☐ MRI-based neuroimaging

Antibodies

Antibodies used

For ICB, each mouse was injected intraperitoneally with 250 ug anti-PD1 (BioXcell, catalog #BE0146, clone #RMP1-14) and 200 ug anti-CTLA-4 (BioXcell, catalog #BE0164, clone 9D9) day 9 and 12 after organoid implantation. For isotype controls, each mouse was injected with 250 ug rat IgG2a (BioXcell, catalog #BE0089, clone 2A3) and 200 ug IgG2b (BioXcell, catalog #BE0086, clone #MPC-11). For CD4+ T cell depletion, each mouse was injected with 250 ug anti-CD4 (BioXcell, catalog #BE0003-1, clone #GK1.5) day 0 and 7 after organoid depletion. Rat IgG2b (BioXcell, catalog #BE0090, clone #LTF-2) was used as isotype control for anti-CD4.

Validation

Each antibody was determined by the manufacturer to be >95% pure by SDS-PAGE.

Eukaryotic cell lines

Policy information about cell lines and Sex and Gender in Research

Cell line source(s)

HCC1395/HCC1395BL from American Type Culture Collection (ATCC), MCB6C cells were thawed from previously archived frozen vial of singly suspended MCB6C organoid

Authentication

The cell lines were not authenticated.

Mycoplasma contamination

All cell lines were confirmed for the absence of mycoplasma.

Commonly misidentified lines
(See ICLAC register)

We do not use commonly misidentified lines.

Animals and other research organisms

Policy information about studies involving animals; ARRIVE guidelines recommended for reporting animal research, and Sex and Gender in Research

Laboratory animals

45 5- to 6-week-old black 6 (B6NTac) male mice were purchased from Taconic Biosciences. The mice were kept under A light:dark cycle of 12 hours on:12 hours off. The animals were housed at humidity 30-70% and temperature 20-26°C

Wild animals

Study did not involve wild animals.

Reporting on sex

Only males were used for this study.

Field-collected samples

Study did not involve samples collected from the field.

Ethics oversight

Under the guidelines of Institutional Animal Care and Use Committee at Washington University (#20-0115).

Note that full information on the approval of the study protocol must also be provided in the manuscript.
